# Benzodiazepine drug use and cancer risk: a dose–response meta analysis of prospective cohort studies

**DOI:** 10.18632/oncotarget.22057

**Published:** 2017-10-19

**Authors:** Tao Zhang, Xiaowen Yang, Jianrui Zhou, Pei Liu, Hui Wang, Anrong Li, Yi Zhou

**Affiliations:** ^1^ Department of Neurosurgery, Taihe Hospital, Hubei University of Medicine, Shiyan, Hubei, 442000, China; ^2^ Department of Clinical Laboratory, Taihe Hospital, Hubei University of Medicine, Shiyan, Hubei, 442000, China; ^3^ Department of Rehabilitation Medicine, Taihe Hospital, Hubei University of Medicine, Shiyan, Hubei, 442000, China; ^4^ Department of Dermatology, Taihe Hospital, Hubei University of Medicine, Shiyan, Hubei, 442000, China

**Keywords:** cancer, benzodiazepine, dose-response relationship, meta analysis

## Abstract

Conflicting results identifying the relationship between benzodiazepine drug use and cancer risk. Therefore, we conducted a dose-response meta-analysis of prospective cohort studies to clarify and quantitative assessed the relationship between benzodiazepine drug use and cancer risk. Up to July 2017, 22 original publications were included in current meta-analysis. Our results showed statistically significant association between benzodiazepine drug use and cancer risk (RR:1.25; 95% CI, 1.15–1.36). Subgroup analysis showed benzodiazepine using was associated with significantly a higher risk of breast cancer (RR:1.15; 95% CI, 1.05–1.26), ovarian cancer (RR:1.17; 95% CI, 1.09–1.25), colon cancer (RR:1.07; 95% CI, 1.02–1.13), renal cancer (RR:1.31; 95% CI, 1.15–1.49), malignant melanoma (RR:1.10; 95% CI, 1.03–1.17), brain cancer (RR:2.06; 95% CI, 1.76–2.43), esophagus cancer (RR:1.55; 95% CI, 1.30–1.85), prostate cancer (RR:1.26; 95% CI, 1.16–1.37), liver cancer (RR:1.22; 95% CI, 1.13–1.31), stomach cancer (RR:1.17; 95% CI, 1.03–1.32), pancreatic cancer (RR:1.39; 95% CI, 1.17–1.64) and lung cancer (RR:1.20; 95% CI, 1.12–1.28). Furthermore, a significant dose-response relationship was observed between benzodiazepine drug use and cancer risk (likelihood ratio test, *P* < 0.001). Our results showed per 500 mg/year, per 5 year of time since first using, per 3 prescriptions and per 3 year of duration incremental increase in benzodiazepine drug use was associated with a 17%, 4%, 16% and 5% in cancer risk increment. Considering these promising results, increasing benzodiazepine using might be harmful for health.

## INTRODUCTION

Cancer is the second most frequently leading cause that caused over 8.8 million deaths worldwide in 2015 [1], and the World Health Organization predicts 14.1 million people are expected to develop cancer annually [2]. Cancer represents a heavy social health problem and economic burden, and costs on patients. The etiology of cancer involves both genetic and environmental factors. Multiple lines of evidence have demonstrated that benzodiazepine drug use is the risk factors for cancer regardless of laboratory studies or animal studies [3, 4].

Benzodiazepines are the derivatives of 1, 4-benzodiazepines, including diazepam, flurazepam, chlorazepam, oxazepam and triazolam. The main function of benzodiazepine is inducing feelings of calm, drowsiness and sleep, and have been widely used in clinical, mainly to treat anxiety and insomnia. Benzodiazepines has been one of the most commonly used drugs in the general population for nearly 50 years, with the use of older people worldwide ranging from 10 to 42 percent. In the United States, about 6 to 10 percent of adults use benzodiazepines in 2010, while in Europe, some regions have found a higher proportion [5, 6]. Therefore, it is important to evaluate the benzodiazepines drugs safety.

Previous studies have examined the correlation between benzodiazepines drug use and cancer risk [7–28]. However, the result remains controversial. Additionally, no study to quantitative assessed benzodiazepines drug use in relation to cancer risk. Thus, we performed this dose-response meta-analysis to clarify and quantitative assessed the correlation between benzodiazepines drug use and cancer risk.

## RESULTS

### Literature search results

**Figure 1:**
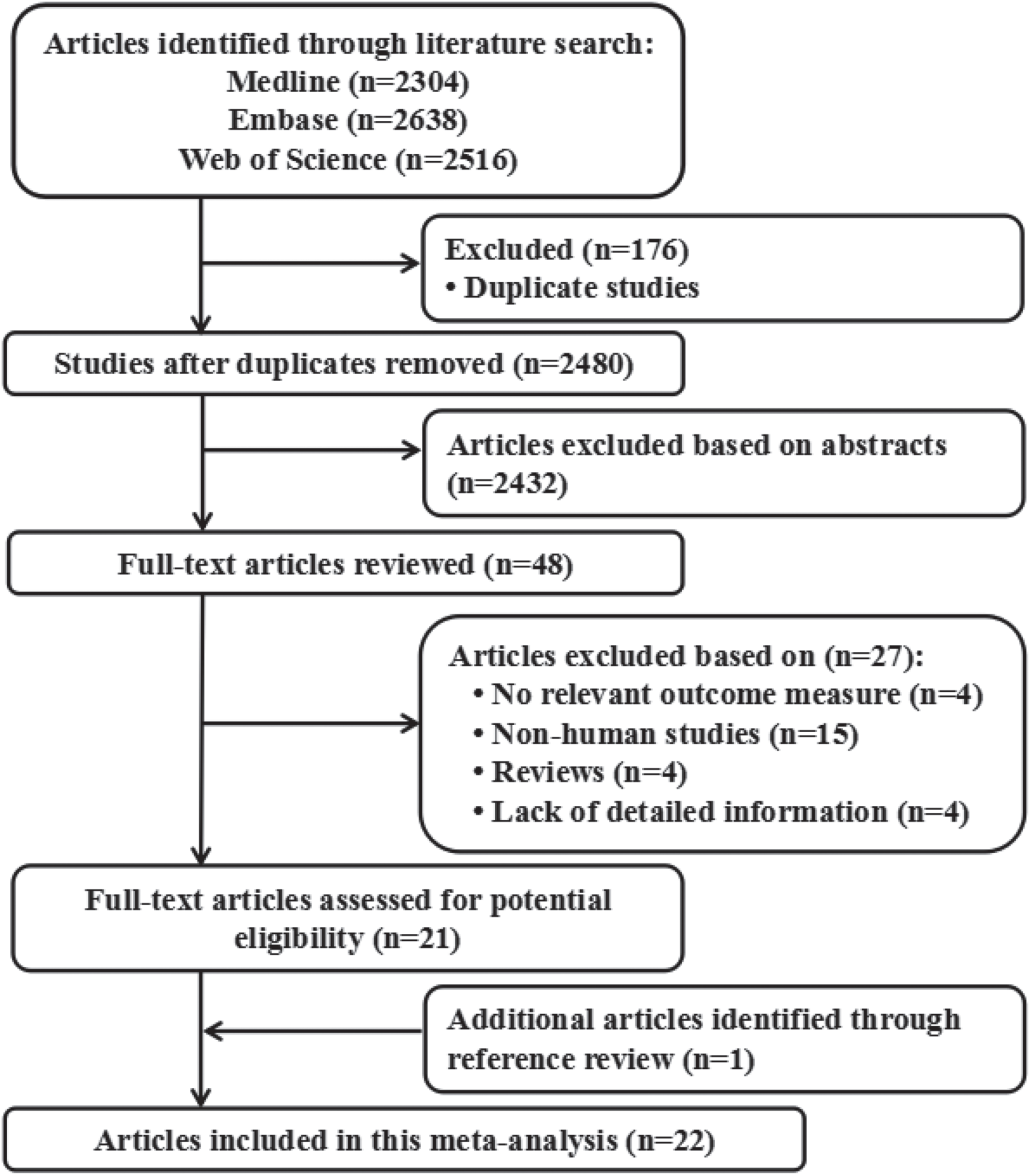
Flow diagram of the study selection process

Figure 1 shows literature research and selection. A total of 2304 studies from PubMed, 2638 studies from Embase and 2516 studies from Web of Science. After exclusion of duplicates and studies that did not fulfill the inclusion criteria, 22 studies were chosen, and the data were extracted. These studies were published update to July 2017.

### Study characteristics

The characteristics of the included studies of benzodiazepines drug use and cancer risk are shown in the Tables 1 and 2. Results in different subgroups were treated as two separate reports. Finally, twenty-seven independent reports from twenty two studies investigated the association between benzodiazepines drug use and cancer risk. Among the selected reports, twenty-two are from Caucasia and five from Asia. A total of 2482625 participants with 312203 incident cases from seven countries were included in this meta-analysis.

**Table 1:** Characteristics of participants in included studies of benzodiazepine using in relation to risk of cancer

Author (year)	Study design	Country	Sex of population	Age at baseline (years)	No of participants	Endpoints (cases)	Quality score
Coogan et al. (2000)	Case-control	United States	Mix	< 69	3740	Ovarian cancer (748)	6
Dublin et al. (2002)	Case-control	United States	Mix	35–79	1104	Ovarian cancer (314)	7
Fortuny et al. (2007)	Case-control	United States	Mix	66.8	4110	Esophageal adenocarcinoma (163) Gastric cardia adenocarcinoma (176) Esophageal squamous cell carcinoma (114) Gastric non-cardia adenocarcinoma (320)	8
Friedman et al. (1998)	Case-control	United States	Mix	30–79	4403	Colon cancer (1960)	7
Halapy et al. (2006)	Case-control	Canada	Female	25–74	6195	Breast cancer (3133)	7
Hardell et al. (1996)	Case-control	Sweden	Mix	NA	987	Colon cancer (329)	5
Harlow et al. (1995)	Case-control	United States	Mix	18–80	904	Ovarian cancer (450)	7
Harnod et al. (2014)	Prospective cohort	China	Mix	≥ 20	62050	Brain cancer (274)	7
Jaussent et al. (2013)	Prospective cohort	France	Mix	65–95	6696	All cancers (1454)	8
Kao et al. (2012)	Prospective cohort	China	Mix	47.9	119239	All cancers (3520)	6
Kaufman et al. (1982)	Case-control	Canada,United States and Israel	Female	< 70	1964	Breast cancer (1236)	6
Kleinerman et al. (1984)	Case-control	United States	Female	≥ 35	2221	Breast cancer (1075)	7
Kaufman et al. (1990)	Case-control	United States Canada	Female	18–69	5009	Breast cancer (3078)	7
Rosenberg et al (1995)	Case-control	United States	Mix	18–69	6077	All cancers (3820)	7
Westerdahl et al. (1996)	Case-control	Sweden	Mix	15–75	1040	Malignant melanoma (400)	7
Lagergren et al. (2000)	Case-control	Sweden	Mix	< 80	1009	Esophageal cancer (189)	7
Pogoda et al. (2005)	Case-control	United States	Mix	25–75	824	Acute myeloid leukemia (412)	7
Lagergren et al. (2006)	Case-control	United States	Female	21–84	870	Malignant melanoma (179)	6
Kripke et al. (2012)	Prospective cohort	United States	Female	≥ 18	25750	All cancers (2076)	8
Pottegardet al. (2012)	Case-control	Denmark	Mix	56–74	1214099	All cancers (149360)	8
Iqbal et al. (2015)	Case-control	China	Mix	≥ 20	255000	All cancers (42500)	8
Thygesen et al. (2017)	Case-control	Denmark	Mix	18–85	759334	All cancers (94923)	7

**Table 2:** Outcomes and covariates of included studies of benzodiazepine using in relation to risk of cancer

Author (year)	Endpoints	Data source	Category and relative risk (95% CI)	Covariates in fully adjusted model
Coogan et al. (2000)	Ovarian cancer (748)	Population-based	Duration of regular use 0 using, 1.0 (reference); 0 to 5 years using, 0.9 (0.6, 1.3); more than 5 years using, 1.1 (0.5, 2.3)	Adjusted for age, study center, and interview year.
Dublin et al. (2002)	Ovarian cancer (314)	Self-administered	Duration of regular use 0 using, 1.0 (reference); 4 months using,0.76 (0.52, 1.10); 6 months using, 0.70 (0.47, 1.0) Number of prescriptions0 prescriptions 1.0 (reference); 1, 0.65 (0.42, 1.0); 2 to 4, 0.91 (0.57, 1.5); > 5, 0.68 (0.42, 1.1)	Adjusted for age and reference date.
Fortuny et al. (2007)	Esophageal adenocarcinoma (163) , Gastric cardia adenocarcinoma (176) Esophageal squamous cell carcinoma (114) Gastric non-cardia adenocarcinoma (320)	Population-based	Esophageal adenocarcinoma Number of prescriptions 0 prescriptions1.0 (reference); < 1, 1.0 (0.5, 1.7); 1to 5, 0.8 (0.5, 1.5); > 5, 0.8 (0.4, 1.5) Gastric cardia adenocarcinoma 0 prescriptions1.0 (reference); < 1, 0.8 (0.5, 1.3); 1to 5, 0.4 (0.1, 0.9); > 5, 0.6 (0.3, 1.1) Esophageal squamous cell carcinoma0 prescriptions 1.0 (reference); < 1, 0.6 (0.3, 1.2); 1to 5, 0.8 (0.4, 1.9); > 5, 1.7 (0.9, 3.1) Gastric non-cardia adenocarcinoma0 prescriptions 1.0 (reference); < 1, 0.6 (0.4,0.8); 1to 5, 0.6 (0.4, 1.0); > 5, 0.5 (0.3, 0.8)	Adjusted for age, sex, HMO, years of enrollment in the HMO, race at HFHS, and adjusted for use of drug classes other than the studied one.
Friedman et al. (1998)	Colon cancer (1960)	Population-based	Duration of regular use 0 using, 1.0 (reference); less than 1years using, 0.9 (0.7, 1.3); 1to 5 years using, 1.2 (0.8, 1.8); more than 5 years using, 0.7 (0.3, 1.2)	Age, sex, aspirin and NSAID use,family history of colorectal cancer,body mass index,total calorie, fiber and calcium intake, physical activity, cigarette smoking and alcohol use
Halapy et al. (2006)	Breast cancer (3133)	Self-administered	Duration of regular use 0 using, 1.0 (reference);less than 1years using, 0.90 (0.66, 1.22); 1to 6 years using, 1.23 (0.90, 1.69); more than 6 years using, 1.32 (0.95, 1.84)	Adjusted for age, family history of breast cancer, and benign breast cysts.
**Jaussent** et al. (2013)	Colon cancer (329)	Population-based	Number of prescriptions 0 prescriptions 1.0 (reference); 1, 0.96 (0.74, 1.25); > 2, 0.93 (0.50, 1.71)	Adjusted for age, study center, and gender; badjusted for all covariates in model 1 plus high level of education, confinement, alcohol intake, smoking status, history of cardio-cerebrovascular disease, respiratory disease, Mini Mental State Examination score, body mass index, hypertension and diabetes mellitus, depressive symptoms, antidepressants use.
**Kaufman** et al. (1982)	Ovarian cancer (450)	Self-administered	Duration of regular use 0 using, 1.0 (reference); less than 5 years using, 0.9(0.4, 1.7); more than 5 years using, 1.0 (0.4, 2.6)	Age, geographical region, years of education, religion, parity, age at first pregnancy, menopausal status, age at menopause, history of breast cancer in the mother or sisters, alcohol consumption, number of visits to a doctor in the preceding year, total number of hospital admissions, and year of interview
Kaufman et al. (1990)	Brain cancer (274)	Population-based	USDuration of regular use 0 using, 1.0 (reference); less than 5 years using, 1.2 ( 0.7, 2.3); more than 5 years using, 0.7 (0.3, 1.6) Canada Duration of regular use 0 using, 1.0 (reference); less than 5 years using, 0.7 (0.4, 1.3); more than 5 years using, 1.0 (0.5, 2)	Age, Alcohol consumption, medical history,lifetime history of medication use, use of muscle relaxants, tranqulizers, psychiatric drugs, insomnia and pain
Lagergren et al. (2000)	All cancers (1454)	Population-based	Duration of regular use 0 using, 1.0 (reference); less than 5 years using, 0.8 (0.6, 3.2); more than 5 years using, 1.5 (0.7, 2.9)	Age, sex, body mass index ,tobacco smoking, alcohol use, socioeconomic status (years of formal education), and intake of fruit and vegetables
**Pottegard** et al. (2012)	All cancers (3520)	Self-administered	Duration of regular use 0 using, 1.0 (reference); less than 1years using, 1.03 (1.02, 1.05); 1to 3 years using, 1.05 (1.02, 1.08); 3to 7 years using, 1.07 (1.03, 1.12); more than 7 years using, 1.11 (1.01, 1.23)	Age, gender, use of aspirin, non-aspirinNSAIDs, 5-areductase inhibitors, statins, angiotensin-II antagonists, oral contraceptives and hormone supplements, antidepressants, antipsychotics, diagnoses of inflammatory bowel disease, COPD, diabetes, alcohol abuse and Charlson Comorbidity Index score

### Benzodiazepines drug use and overall cancer risk

Twenty-seven independent reports from twenty two studies investigated the association between between benzodiazepines drug use and cancer risk. Compared with no benzodiazepines drug use, benzodiazepines drug use is significantly associated with a higher risk of cancer risk (RR:1.25; 95% CI, 1.15–1.36; *P* < 0.001) (Table 3). Furthermore, benzodiazepines drug use is significantly associated with cancer risk in Caucasia (RR:1.21; 95% CI, 1.05–1.39; *P* < 0.001) (Table 3) and Asia (RR:1.36; 95% CI, 1.16–1.59; *P* < 0.001) (Table 3). Additionally, benzodiazepines drug use is significantly associated with cancer risk in female (RR:1.14; 95% CI, 1.04–1.24; *P* = 0.004) (Table 3) but not in male (RR:1.12; 95% CI, 0.96–1.30; *P* = 0.154) (Table 3). That may be because there isn't enough data in male.

**Table 3:** Stratified analyses of relative risk of cancer

	No of reports	Relative risk (95% CI)	P for heterogeneity	I^2^	*P* for test
Total	27	1.25 (1.15–1.36)	0.000	78.8%	< 0.001
Subgroup analyses for cancer					
Study location					
Caucasia	22	1.21 (1.05–1.39)	0.000	66.3%	0.008
Asia	5	1.36 (1.16–1.59)	0.000	91.0%	< 0.001
Gender					
Female	10	1.14 (1.04–1.24)	0.015	56.2%	0.004
Male	2	1.12 (0.96–1.30)	0.591	0.0%	0.154
Study design					
Case–control	21	1.15 (1.05–1.26)	0.000	61.5%	0.002
Cohort	6	1.43 (1.12–1.83)	0.000	92.0%	< 0.001
Study quality					
Score ≥ 7	20	1.27 (1.13–1.42)	0.000	84.0%	< 0.001
Score < 7	7	1.20 (1.12–1.29)	0.484	0.0%	< 0.001
No of participants					
≥ 10 000	7	1.43 (1.12–1.83)	0.000	92.0%	< 0.001
< 10 000	20	1.15 (1.05–1.26)	0.000	61.5%	0.002
No of cases					
≥ 1000	13	1.26 (1.16–1.37)	0.000	79.5%	< 0.001
< 1000	14	1.22 (1.13–1.31)	0.000	62.9%	< 0.001
Types of benzodiazepine					
Long-acting (Diazepam)	8	1.08 (0.94–1.24)	0.075	50.1%	0.306
Intermediate-acting	6	1.21 (1.16–1.23)	0.000	75.3%	< 0.001
Short-acting	3	1.16 (1.07–1.26)	0.671	0.0%	< 0.001
Duration of benzodiazepine use					
0 years	8	1			
< 5 years	8	1.09 (1.05–1.14)	0.000	64.6%	< 0.001
≥ 5 years	8	1.20 (1.16–1.23)	0.000	72.8%	< 0.001
Cumulative yearly dose					
Lower	3	1			
Moderate	3	1.59 (1.26–2.00)	0.000	62.8%	< 0.001
Highest	3	2.93 (2.45–3.52)	0.000	96.6%	< 0.001
Number of prescriptions					
Lower	6	1			
Highest	6	1.12 (1.03–1.22)	0.069	51.2%	< 0.001
Time since first use					
0 years	5	1			
< 10 years	5	1.14 (1.05–1.24)	0.753	0.0%	< 0.001
≥ 10 years	5	1.23 (1.13–1.33)	0.175	36.9%	< 0.001
Time since last benzodiazpine use					
0 years	2	1			
< 1 years	2	0.97 (0.79–1.20)	0.381	0.0%	0.781
≥ 1 years	2	1.16 (0.87–1.56)	0.889	0.0%	0.322

### Benzodiazepine drug use and the risk of cancer by type of cancer

Eleven independent reports from seven studies investigated the association between benzodiazepines drug use and breast cancer. Compared with no benzodiazepines drug use, benzodiazepines drug use is significantly associated with a higher risk of breast cancer (RR:1.15; 95% CI, 1.05–1.26; *P* < 0.001) (Table 4). Furthermore, benzodiazepines drug use is significantly associated with breast cancer risk in Caucasia (RR:1.17; 95% CI, 1.08–1.26; *P* < 0.001) (Table 4) and Asia (RR:1.09; 95% CI, 1.03–1.16; *P* < 0.001) (Table 4). Eight independent reports from six studies investigated the association between benzodiazepines drug use and ovarian cancer risk. Compared with no benzodiazepines drug use, benzodiazepines drug use is significantly associated with a higher risk of ovarian cancer (RR:1.17; 95% CI, 1.09–1.25; *P* < 0.001) (Table 4). Furthermore, benzodiazepines drug use is significantly associated with ovarian cancer risk in Caucasia (RR:1.22; 95% CI, 1.15–1.30; *P* < 0.001) (Table 4) and Asia (RR:1.11; 95% CI, 1.05–1.17; *P* < 0.001) (Table 4). Additionally, Compared with no benzodiazepines drug use, benzodiazepines drug use is significantly associated with a higher risk of colon cancer (RR:1.07; 95% CI, 1.02–1.13; *P* < 0.001) (Table 4), renal cancer (RR:1.31; 95% CI, 1.15–1.49; *P* < 0.001) (Table 4), malignant melanoma (RR:1.10; 95% CI, 1.03–1.17; *P* < 0.001) (Table 4), brain cancer (RR:2.06; 95% CI, 1.76–2.43; *P* < 0.001) (Table 4), esophagus cancer (RR:1.55; 95% CI, 1.30–1.85; *P* < 0.001) (Table 4), prostate cancer (RR:1.26; 95% CI, 1.16–1.37; *P* < 0.001) (Table 4), liver cancer (RR:1.22; 95% CI, 1.13–1.31; *P* < 0.001) (Table 4), stomach cancer (RR:1.17; 95% CI, 1.03–1.32; *P* < 0.001) (Table 4), pancreatic cancer (RR:1.39; 95% CI, 1.17–1.64; *P* < 0.001) (Table 4), lung cancer (RR:1.20; 95% CI, 1.12–1.28; *P* < 0.001) (Table 4).

**Table 4:** Stratified analyses of relative risk of different cancer

	No of reports	Relative risk (95% CI)	P for heterogeneity	I^2^	*P* for test
Breast cancer	11	1.15 (1.05–1.25)	0.345	17.3%	< 0.001
Subgroup analyses for Breast cancer					
Study location					
Caucasia	7	1.17 (1.08–1.26)	0.258	25.6%	< 0.001
Asia	4	1.09 (1.03–1.16)	0.631	0.0%	< 0.001
Study design					
Case–control	8	1.05 (1.01–1.09)	0.214	23.6%	< 0.001
Cohort	3	1.19 (1.12–1.28)	0.447	0.0%	< 0.001
Study quality					
Score ≥ 7	7	1.06 (1.02–1.10)	0.215	24.3%	< 0.001
Score < 7	4	1.19 (1.11–1.28)	0.474	0.0%	< 0.001
No of participants					
≥ 10 000	8	1.05 (1.01–1.09)	0.214	23.6%	< 0.001
< 10 000	3	1.19 (1.12–1.28)	0.447	0.0%	< 0.001
No of cases					
≥ 1500	9	1.07 (1.02–1.12)	0.101	33.6%	< 0.001
< 1500	2	1.18 (1.10–1.26)	0.673	0.0%	< 0.001
Ovarian cancer	8	1.17 (1.09–1.25)	0.000	74.5%	< 0.001
Subgroup analyses for Ovarian cancer					
Study location					
Caucasia	4	1.22 (1.15–1.30)	0.008	80.2%	< 0.001
Asia	4	1.11 (1.05–1.17)	0.657	0.0%	< 0.001
Study design					
Case–control	5	1.14 (1.02–1.28)	0.000	84.4%	0.012
Cohort	3	1.19 (1.12–1.28)	0.447	0.0%	< 0.001
Study quality					
Score ≥ 7	3	1.19 (1.12–1.28)	0.447	0.0%	< 0.001
Score < 7	5	1.14 (1.02–1.28)	0.000	84.4%	0.012
No of participants					
≥ 10 000	3	1.19 (1.12–1.28)	0.447	0.0%	< 0.001
< 10 000	5	1.14 (1.02–1.28)	0.000	84.4%	0.012
No of cases					
≥ 1500	3	1.19 (1.12–1.28)	0.447	0.0%	< 0.001
< 1500	5	1.14 (1.02–1.28)	0.000	84.4%	0.012
Colon cancer	4	1.07 (1.02–1.13)	0.361	10.4%	< 0.001
Renal cancer	4	1.31 (1.15–1.49)	0.198	25.7%	< 0.001
Malignant melanoma	3	1.10 (1.03–1.17)	0.715	0.0%	< 0.001
Brain cancer	4	2.06 (1.76–2.43)	0.000	86.1%	< 0.001
Esophagus cancer	4	1.55 (1.30–1.85)	0.679	0.0%	< 0.001
Prostate cancer	3	1.26 (1.16–1.37)	0.000	79.5%	< 0.001
Liver cancer	3	1.22 (1.13–1.31)	0.000	62.9%	< 0.001
Stomach cancer	2	1.17 (1.03–1.32)	0.174	45.6%	< 0.001
Pancreatic cancer	2	1.39 (1.17–1.64)	0.813	0.0%	< 0.001
Lung cancer	3	1.20 (1.12–1.28)	0.000	89.8%	< 0.001

### Subgroup analyses

Subgroup analysis was performed to check the stability of the primary outcome. Subgroup meta-analyses in study design, study quality, number of participants and number of cases showed consistent findings (Tables 3, 4).

### Dose-response between benzodiazepines drug use and cancer risk

**Figure 2:**
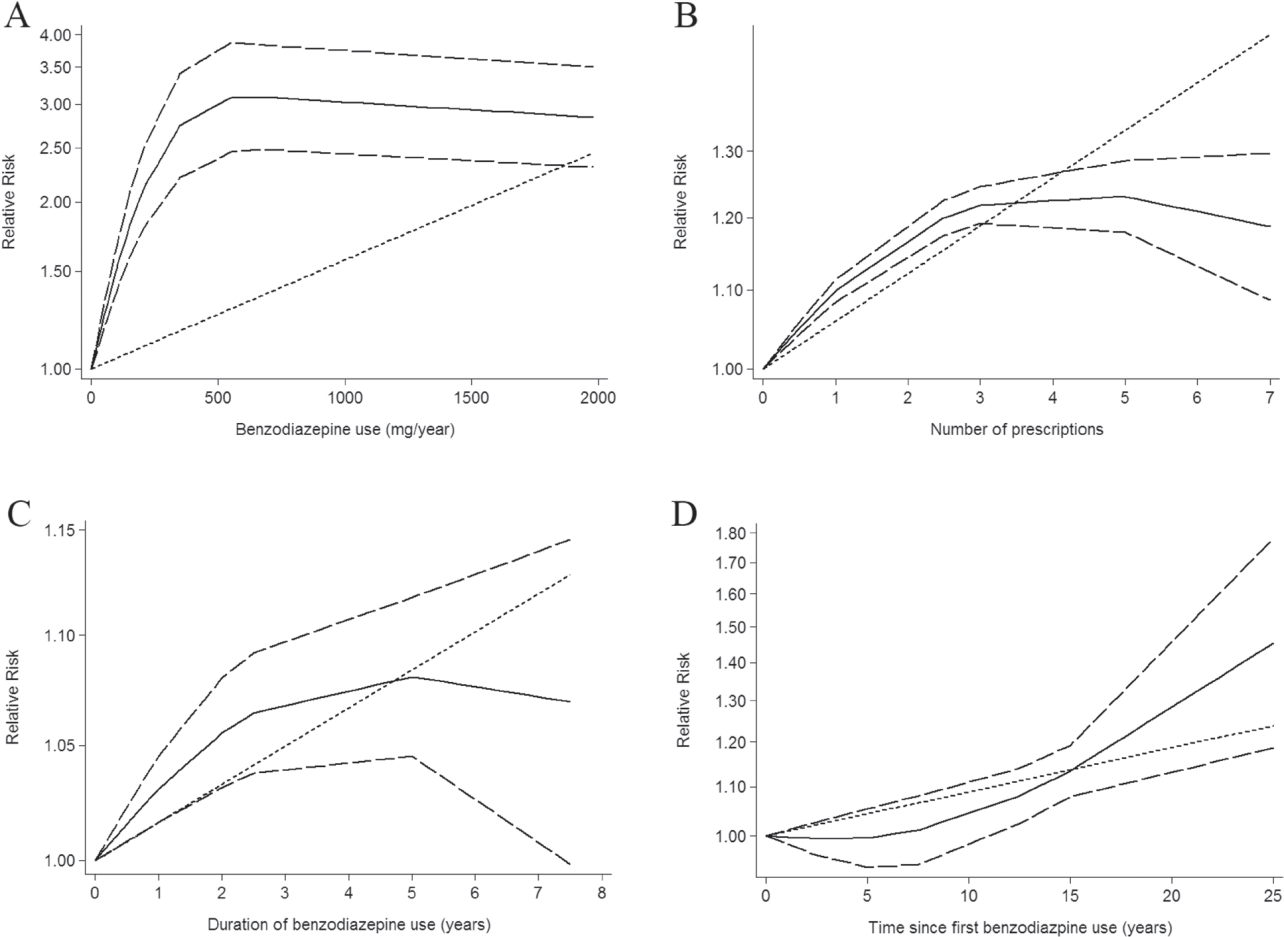
Dose-response relationship between benzodiazepine drug use in relation to risk of cancer (**A**) Cumulative yearly dose of benzodiazepine drug use. (**B**) Number of prescriptions benzodiazepine drug use. (**C**) Duration of benzodiazepine drug use. (**D**) Time since first benzodiazepine drug use.

Using restricted cubic spline function, the test for a nonlinear dose-response relationship was significant (likelihood ratio test, *P* < 0.001), suggesting curvature in the relationship, increasing per 500 mg/year of benzodiazepine drug use was associated with a 17% increment of cancer risk, the summary relative risk of cancer risk for an per 500 mg/year of benzodiazepine drug use was 1.17 (95% CI: 1.02–1.35, *P* = 0.022) (Figure 2). In addition, increasing benzodiazepine drug use (per 3 prescriptions increment) was associated with a 16% increment in cancer risk, the summary relative risk of cancer risk for an per 3 prescriptions of benzodiazepine drug use was 1.16 (95% CI: 1.11–1.22, *P* < 0.001) (Figure 2). Furthermore, increasing per 3 year of duration of benzodiazepine drug use was associated with a 5% increment of cancer risk, the summary relative risk of cancer risk for an per 3 year of duration of benzodiazepine drug use was 1.05 (95% CI: 1.02–1.09, *P* = 0.042) (Figure 2). Additionally, increasing per 5 year of time since first benzodiazepine drug use was associated with a 4% increment of cancer risk, the summary relative risk of cancer risk for an per 5 year of time since first benzodiazepine drug use was 1.04 (95% CI: 1.01–1.07, *P* = 0.003) (Figure 2).

### Sensitivity analysis

Sensitivity analysis was conducted to assess the stability of the results. The results show the results were stable in Supplementary Figure 1.

### Publication bias

Each studies in this meta-analysis were performed to evaluate the publication bias by both Begg's funnel plot and Egger's test. *P* > 0.05 was considered no publication bias. The results show no obvious evidence of publication bias was found in the associations between benzodiazepine drug use and cancer risk (Supplementary Table 1).

## DISCUSSION

Benzodiazepines are widely used to treat seizures, anxiety, insomnia, and panic disorder. In recent years there has been constant evidence that benzodiazepines are associated with cancer risk *in vitro* laboratory and animal studies. Several animal studies reported that benzodiazepines increase the risk of thyroid cancer [29] or liver cancer [3] through inhibiting apoptosis and stimulating tumor cell proliferation. In the meantime, several observational epidemiological studies reported that there was no link between benzodiazepines drug use and the risk cancer. However, recent cohort studies revealed that the use of benzodiazepines drugs was associated with an increased risk of cancer, and the benzodiazepine users were exposed to the risk of benign brain tumor about three times higher than the non-benzodiazepine users. Collectively, these data suggest that benzodiazepines drug use may play an important role in cancer risk, but presented controversial results. Thus, we performed this meta-analysis, aiming to study the role of benzodiazepines in cancer risk and to explain the possible reasons for controversial results.

In the current meta-analysis was based on 22 case-control or cohort study, with 2482625 participants with 312203 incident cases from seven countries. Thus, this meta analysis provides the most up-to-date epidemiological evidence supporting benzodiazepines drug use is harmful for health. A dose-response analysis revealed that increasing per 500 mg/year of benzodiazepine drug use was associated with a 17% increment of cancer risk. In addition, increasing benzodiazepine drug use (per 3 prescriptions increment) was associated with a 16% increment in cancer risk. Furthermore, increasing per 3 year of duration of benzodiazepine drug use was associated with a 5% increment of cancer risk. Additionally, increasing per 5 year of time since first benzodiazepine drug use was associated with a 4% increment of cancer risk. Subgroup meta-analyses by various factors also showed consistent findings.

Several plausible pathways may reasonable for the relationship between benzodiazepine drug use and cancer risk. The influence of chronic inflammation on cancer development is one possible pathway. Benzodiazepine drug use might increase the levels of inflammation mediators [30], which can increase the risk of cancer by inhibiting apoptosis and stimulating tumor cell proliferation [31]. Simultaneously, the treatment of benzodiazepine drug use before surgery can influence the depolarization of the mitochondrial membrane to inhibit the apoptosis of neutrophil cells, neutrophil apoptosis plays an important role in maintaining the homeostasis of the immune system and prevents damage to the host organs by promoting an immune response [32]. Addition, benzodiazepines are used to enhance the neurotransmitter of γ-aminobutyric acid by interacting with the chlorine ion channel that binds to GABA receptors. Besides as an inhibitory neurotransmitter effect, γ-aminobutyric acid is also thought can regulate cell proliferation and differentiation of brain and peripheral at various stages, and may participate in benign tumor growth in a variety of ways [33, 34]. However, the potential mechanisms of benzodiazepines and tumor growth still remain unclear and controversial.

To our knowledge, this is the first study to identify and quantify the potential dose-response association between benzodiazepine drug use and cancer risk in a large cohort of both men and women. Although, we performed this meta-analysis very carefully, however, some limitations must be considered in the current meta-analysis. First, different sex of population should be included in this meta-analysis to explore the impact of different sex of population on benzodiazepine drug use and cancer risk. Second, we only select literature that written by English, which may have resulted in a language or cultural bias, other language should be chosen in the further. Third, in the subgroup analysis in cancer type, there has no insufficient statistical power to check a dose-response in different cancer type, large data in different cancer type is warranted to validate this association.

In conclusion, our findings underscore the notion that benzodiazepine drug use was significantly associated with cancer risk increment. In the future, large-scale and population based association studies must be performed in the future to validate the risk identified in the current meta-analysis.

## MATERIALS AND METHODS

This meta-analysis was conducted according to the Meta-analysis Of Observational Studies in Epidemiology (MOOSE) checklist [35].

### Search strategy

We included eligible studies to investigate the relationship between benzodiazepines drug use and cancer risk. To develop a flexible, non-linear, r meta-regression model, we required that an eligible study should have categorized into 3 or more levels.

PubMed, EMBASE and Web of Science databases were searched for studies that contained risk estimates for the outcomes of cancer and were published update to July 2017, with keywords including “benzodiazepine” [MeSH] OR ”diazepam” [MeSH] OR “alprazolam” [MeSH] OR “clonazepam” [MeSH] OR “temazepam” [MeSH] OR “oxazepam” [MeSH] AND “cancer” [MeSH] OR “tumor” [MeSH] “carcinoma” [MeSH] “neoplasm” [MeSH]. We refer to the relevant original essays and commentary articles to determine further relevant research.

### Study selection

Two independent researchers investigate information the correlation between benzodiazepines drug use and cancer risk: outcome was cancer. Moreover, we precluded non-human studies, reviews, meta-analyses, editorials and published letters. To ensure the correct identification of qualified research, the two researchers read the reports independently.

### Data extraction

Use standardized data collection tables to extract data. Each eligible article information was extracted by two independent researchers. We extracted the following information: first author; publication year; age; country; sex; cases and participants; the categories of benzodiazepines drug use; the relative risk or odds ratio (OR). We collect the risk estimates with multivariable-adjusted. According to the Newcastle-Ottawa scale, quality assessment was performed for non-randomized studies [36].

### Statistical analysis

We pooled relative risk estimates to measure the association between benzodiazepines drug use and cancer; the hazard ratio were considered equivalent to the relative risk [37]. Results in different subgroups of benzodiazepines drug use and cancer risk were treated as two separate reports.

Due to different definitions cut-off points in the included studies for categories, we performed a relative risk estimates by increaseper 500mg/year of benzodiazepine using or per 3 prescriptions increment or per 3 year of duration of benzodiazepine using or per 5 year of time since first benzodiazepine use using the method recommended by Greenland, Longnecker and Orsini and colleagues. In addition, using restricted cubic splines to evaluate the non-linear association between benzodiazepines drug use and cancer risk, with three knots at the 10th, 50th, and 90th percentiles of the distribution. A flexible meta-regression based on restricted cubic spline (RCS) function was used to fit the potential non-linear trend, and generalized least-square method was used to estimate the parameters. This procedure treats benzodiazepines drug use (continuous data) as an independent variable and logRR of diseases as a dependent variable, with both tails of the curve restricted to linear. A *P* value is calculated for linear or non-linear by testing the null hypothesis that the coefficient of the second spline is equal to zero [38].

We use STATA software 12.0 (STATA Corp, College Station, TX, USA) to evaluate the relationships between benzodiazepines drug use and cancer risk. By using Q test and I^2^ statistic to assess heterogeneity among studies. Random-effect model was chosen if *P*_Q_< 0.10 or I^2^ > 50%, otherwise, fixed-effect mode was applied. Sensitivity analysis was conducted to assess the stability of the results. Begg's and Egger's tests were to assess the publication bias of each study. *P* < 0.05 was considered signifcant for all tests.

## SUPPLEMENTARY MATERIALS FIGURES AND TABLES


