# Supplementary material for: Benzodiazepine drug use and cancer risk: a dose–response meta analysis of prospective cohort studies
Source: Oncotarget. 2017 Oct 19;8(60):102381–91. doi: 10.18632/oncotarget.22057 (PMC5731963; doi:10.18632/oncotarget.22057)
Supplement: Supplementary file 1 [file oncotarget-08-102381-s001.pdf]

Benzodiazepine drug use and cancer risk: a dose–response meta analysis of prospective cohort studies

SUPPLEMENTARY MATERIALS

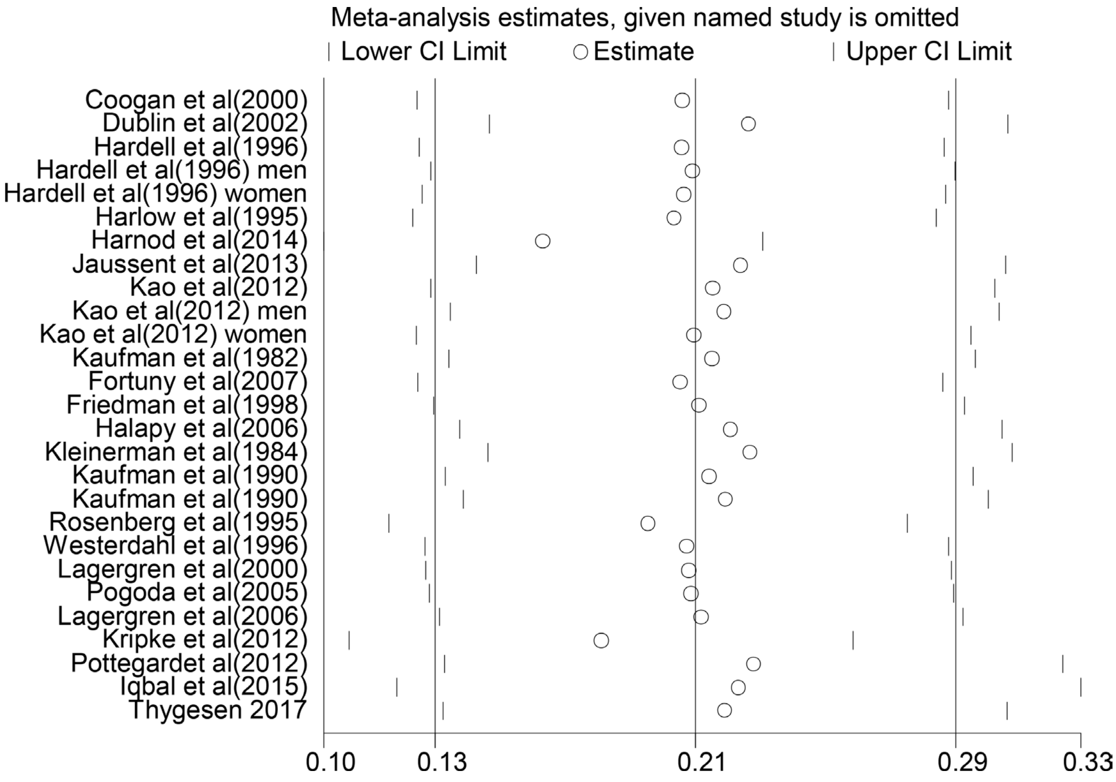

Supplementary Figure 1: Sensitivity analysis of the meta-analysis.

Supplementary Table 1: Publication bias analysis of the meta-analysis

	Test	*t*	95% CI	*P*
**Overall Cancer**	Begg's test			0.588
	Egger's test	0.73	−0.66, 1.39	0.473
**Breast cancer**	Begg's test			0.930
	Egger's test	1.26	−2.97, 2.87	0.863
**Ovarian cancer**	Begg's test			0.118
	Egger's test	0.90	−1.24, 2.79	0.401
**Colon cancer**	Begg's test			0.327
	Egger's test	0.72	−1.52, 3.11	0.487
**Renal cancer**	Begg's test			0.360
	Egger's test	−1.79	−2.83, 0.28	0.099
**Malignant melanoma**	Begg's test			1.000
	Egger's test	−0.34	−7.93, 6.46	0.764
**Brain cancer**	Begg's test			0.917
	Egger's test	0.26	−1.86, 2.30	0.809
**Esophagus cancer**	Begg's test			0.128
	Egger's test	1.13	−0.27, 1.04	0.267
**Liver cancer**	Begg's test			0.472
	Egger's test	−3.27	−5.21, 1.11	0.086
**Lung cancer**	Begg's test			0.791
	Egger's test	−1.74	−0.48, 6.99	0.403
